# Concomitant Vaginal Laceration and Urinary Bladder Injury With Pubic Diastasis: A Case Report on a Rare Complication During Obstructed Labor

**DOI:** 10.7759/cureus.33900

**Published:** 2023-01-17

**Authors:** Gaurav Kumar Malvi, Sujata K Patwardhan, Mayank Agrawal, Rajvi Goradia, Abhinav Malvi

**Affiliations:** 1 Urology, Seth Gordhandas Sunderdas Medical College (GSMC) and the King Edward Memorial (KEM) Hospital, Mumbai, IND; 2 Surgery, Jawaharlal Nehru Medical College, Datta Meghe Institute of Higher Education and Research, Wardha, IND

**Keywords:** primi-gravidae, vaginal injury, urinary bladder perforation, pubic symphysis diastasis, labor complication

## Abstract

A serious uro-obstetric emergency is the concurrent rupture of the uterine and urine bladder following a protracted difficult delivery. In the absence of circumstances that would make the bladder more likely to cling to the lower uterine segment, the involvement of the urinary bladder in a primigravida is unique and relatively infrequent. We discuss a case of a 21-year-old patient who had an obstructed labor complicated with bladder and vaginal injury. At laparotomy, we found a pubic bone diastasis, a vaginal injury, and a bladder injury at the urethrovesical junction. As a result, bladder neck repair with urethrovesical anastomosis and vaginal repair with an external fixator were carried out for pubic bone diastasis.

## Introduction

A serious uro-obstetric emergency that can result in stillbirth, maternal illness, or even maternal death that occurs during labor and delivery is when the bladder ruptures within the uterus [1]. Uterine rupture seldom occurs in a primigravida. In the few isolated cases of uterine rupture due to prolonged, difficult labor in primigravida that has been described, bladder rupture is noticeably rare [2,3]. Obstructed and ignored labor consequences include fetal death, shock, sepsis, uterine rupture, urinary bladder rupture, genitourinary fistula, and recto-uterine fistula. We describe a case of obstructed labor that resulted in urogenital trauma and pubic bone injury. 

## Case presentation

The patient was a twenty-one-year-old female primigravida with nine-month amenorrhea who was referred to the local hospital because of eclampsia. She had one episode of convulsion. When the patient came to the local hospital, she was brought with the baby’s head outside the vagina and her shoulder stuck inside the vagina (shoulder dystocia). Episiotomy was given, and the baby was delivered. The placenta was delivered, and the uterus was well contracted. The patient had a vaginal tear with bladder injury and dribbling of urine. So she was referred to the urology center for further management. 

Examination 

**Figure 1:**
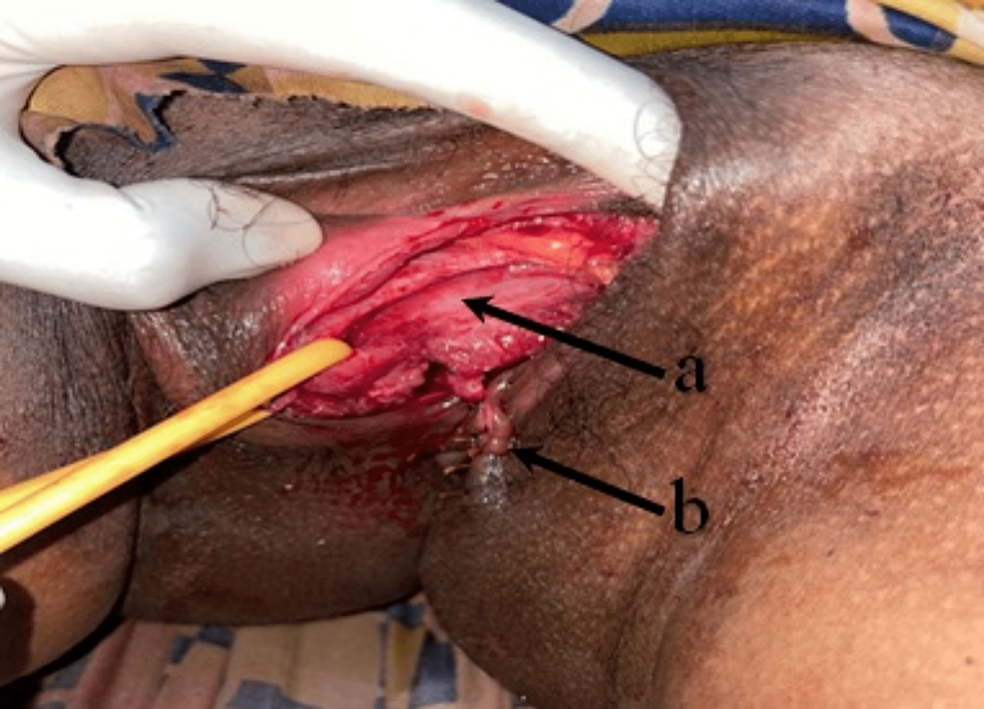
Picture showing (a) bladder tear with Foley’s catheter and (b) episiotomy wound

General condition was poor. Her vitals were: pulse 116/min and blood pressure 86/58 mmHg. Pallor was present. Episiotomy wound was visible. The urethra could not be delineated. Anterior vaginal wall laceration was present (Figure 1). On digital rectal examination, no evidence of rectal injury or blood on the gloved finger was found. 

Surgery 

The patient underwent emergency exploration for bladder neck repair and bladder perforation repair with urethrovesical anastomosis, vaginal wall repair with suprapubic catheter insertion, and external fixation for pubic diastasis. 

Intraoperative findings 

**Figure 2:**
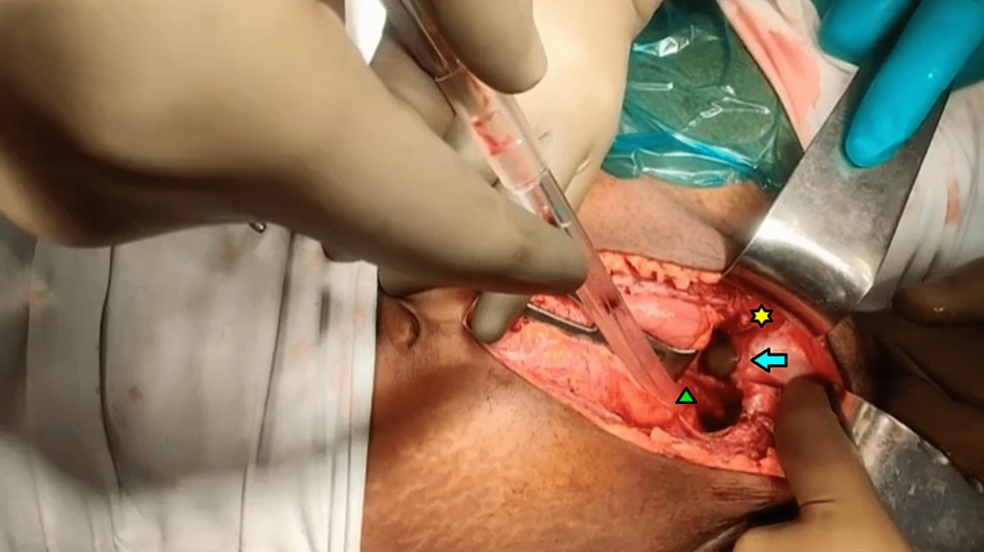
Intraoperative picture showing urinary bladder injury (green arrowhead) with vaginal laceration (finger inserted per vaginally seen coming into the wound as shown by the blue arrow), and pubic bone diastasis (yellow star).

**Figure 3:**
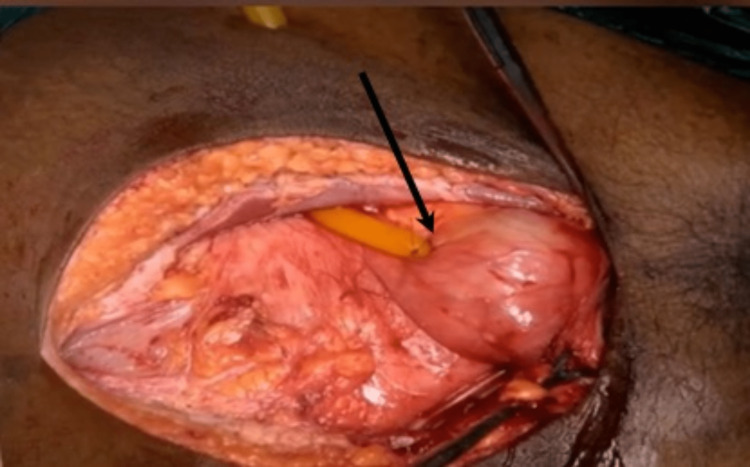
Intraoperative picture showing bladder repair with a supra-pubic catheter (arrow)

The bladder was open with around a 6cm to 8cm tear. Anterior vaginal wall laceration and pubic diastasis of around 2cm to 3cm were present (Figures 2, 3). 

Perioperative course

The duration of the surgery was 3:30 hours. The patient was shifted to the ICU with a supra-pubic catheter and per-urethral catheter, and an abdominal drain was kept in situ. The patient was kept on inotropic support and two packed cell volumes of blood were transfused. 

Postoperative course

The patient was vitally improved and inotropes were withheld on postoperative day (POD) 4. The abdominal drain was removed on POD 10. The patient was discharged on POD 12 with a per-urethral catheter, supra-pubic catheter, and external pubic bone fixator in situ. The per-urethral catheter was removed after six weeks and the supra-pubic catheter after seven weeks of surgery. The orthopedic team removed external fixators for pubic bone after two months. 

## Discussion

With a case fatality rate of 87% to 100%, obstructed labor is one of the most significant causes of perinatal mortality as well as the primary cause of maternal death [4,5]. Because it is such a rare occurrence, it is uncertain whether total urethrovesical disruption happens during obstructed labor. Only two patients (an incidence of 1.9%) of the 102 cases of obstructed labor that were discovered in a retrospective analysis of our patients from 2004 to 2009 had bladder/urethral injuries [6].

Because it is situated so close to the female reproductive system, the lower urinary system is susceptible to injury during uterine rupture. The combination of a ruptured uterus during birth and a damaged bladder has, however, only rarely been reported [7]. An extensive bladder-involved uterine rupture made the patient's protracted, tough labor even more difficult. Urinary bladder involvement in uterine rupture is infrequent in the absence of urinary bladder adhesions to the lower uterine segment, uncommon in primigravida, and uncommon in uteri with no scarring [8].

This particular labor obstruction was probably caused by an inadequate pelvis. The urethrovesical junction may have been damaged and the vagina lacerated as a result of prolonged labor and sustained pressure from the fetal head [9]. Ischemia may have been brought on by these factors and other debilitating factors.

The clinical signs of concurrent uterine and bladder rupture at the moment of vaginal birth depend on the timing, location, and kind of uterine rupture that extends into the adjacent organs. A complete rupture might cause vaginal bleeding, abdominal pain, a troubling fetal cardiac trace, a receding portion on pelvic inspection, a change in uterine shape, cessation of uterine contractions, and even maternal shock [10,11].

The management of female patients with urethral injuries is not standardized. Urinary diversion with a delayed repair is recommended by one school of thought [12], whereas a different school advocates the main final repair at the time of first damage, maybe with the possible exception of severe urethral disruption, which may call for challenging plastic procedures. In a prospective case series of 25 patients, Roenneburg et al. assessed the effectiveness of direct reanastomosis of the remaining distal urethra to the urethro-vesical junction as the major strategy of repair in the traumatic absence of the proximal urethra [12]. They concluded that direct layered reanastomosis is a suitable surgery in such circumstances, however, it is connected to problems like urethral incontinence and anastomotic site leaking. Primary end-to-end anastomosis has a 52% failure rate and a 48% dry rate, according to the same study, and 35% of patients required sling surgery as a second stage of surgery for urethral incontinence [12].

Our patient's poor health-seeking behavior, unfavorable obstetric history, and poor pregnancy outcome are all established risk factors that were influenced by her inadequate pregnancy knowledge, lack of formal education, and low socioeconomic level. The patient displayed clinical indicators of uterine rupture with bladder involvement [1,2,5]. She was consequently quickly revived, assessed, and underwent a laparotomy to be handled as an uro-obstetric emergency with an orthopedic problem [1].

The diagnosis was validated at the laparotomy. Fortunately, the patient's rehabilitation went smoothly before she was released and throughout her subsequent follow-up sessions. She was also told that all following pregnancies, labors, and deliveries should be attended by medical professionals and that only high-risk deliveries should be performed in facilities equipped for such situations.

Any improvement must first begin with a broad improvement in the socioeconomic environment as a whole followed by easier access to healthcare services, better medical center equipment, and retraining of medical staff. Education of women and the broader public could significantly lessen such instances of uterine rupture [13].

## Conclusions

Bladder wall rupture during spontaneous vaginal birth is an extremely unusual event, often linked to obstructed labor. Because uterine rupture and urinary bladder involvement are uncommon among primigravidas, a high index of suspicion helps with therapy. Good antenatal care, family planning services, prompt referral of obstructed labor, accessibility to transportation, and obstetric treatment are essential for preventing uterine rupture and lowering the associated maternal mortality, fetal death, and maternal morbidity.

The effects of morbidities and occasional mortality are lessened by quick and effective intervention. Campaigns to raise public awareness of the dangers of teenage pregnancy, risky obstetric practices, unattended pregnancies, labor, and births are necessary. Stricter rules may result in greater compliance. This case is reported due to its uniqueness and to explore the best treatment option in such instances.
